# Experts bodies, experts minds: How physical and mental training shape the brain

**DOI:** 10.3389/fnhum.2014.00280

**Published:** 2014-05-07

**Authors:** Ursula Debarnot, Marco Sperduti, Franck Di Rienzo, Aymeric Guillot

**Affiliations:** ^1^Département des Neurosciences Fondamentales, Centre Médical Universitaire, Université de GenéveGenéve, Suisse; ^2^Centre de Recherche et d’Innovation sur le Sport, Université Claude Bernard Lyon 1, Université de Lyon, Villeurbanne CedexLyon, France; ^3^Centre de Psychiatrie et Neurosciences (Inserm UMR S894), Université Paris DescartesParis, France; ^4^Laboratoire Mémoire et Cognition, Institut de PsychologieBoulogne-Billancourt, France; ^5^Institut Universitaire de FranceParis, France

**Keywords:** expertise, motor skill, motor imagery, meditation, motor consolidation, neural networks

## Abstract

Skill learning is the improvement in perceptual, cognitive, or motor performance following practice. Expert performance levels can be achieved with well-organized knowledge, using sophisticated and specific mental representations and cognitive processing, applying automatic sequences quickly and efficiently, being able to deal with large amounts of information, and many other challenging task demands and situations that otherwise paralyze the performance of novices. The neural reorganizations that occur with expertise reflect the optimization of the neurocognitive resources to deal with the complex computational load needed to achieve peak performance. As such, capitalizing on neuronal plasticity, brain modifications take place over time-practice and during the consolidation process. One major challenge is to investigate the neural substrates and cognitive mechanisms engaged in expertise, and to define “expertise” from its neural and cognitive underpinnings. Recent insights showed that many brain structures are recruited during task performance, but only activity in regions related to domain-specific knowledge distinguishes experts from novices. The present review focuses on three expertise domains placed across a motor to mental gradient of skill learning: sequential motor skill, mental simulation of the movement (motor imagery), and meditation as a paradigmatic example of “pure” mental training. We first describe results on each specific domain from the initial skill acquisition to expert performance, including recent results on the corresponding underlying neural mechanisms. We then discuss differences and similarities between these domains with the aim to identify the highlights of the neurocognitive processes underpinning expertise, and conclude with suggestions for future research.

Brain plasticity refers to the putative changes in neural organization that accounts for the diverse forms of short-lasting or enduring behavioral modifiability. There is considerable evidence that neuronal plasticity is not an occasional state, but rather allows the human brain to adapt to environmental pressure, physiologic changes, and experiences (Johansson, 2004). Interestingly, changes in the input of any neural system, or in the trafficking of its efferent connections, lead to reorganizations that are visible at the level of behavior, anatomy, and physiology, encompassing cellular and molecular levels (Pascual-Leone et al., 2005). Currently, the challenge is to explore in greater details the processes of neuroplasticity and how to modulate them to achieve the best behavioral outcome. Typically, the human brain undergoes constant changes triggered by environmental stimulation or resulting from intrinsic remodeling activity (Johansson, 2011). Therefore, brain is the source of behavior, but in turn is modified by the behavior itself.

Most of the prior studies that aimed to investigate the neural substrates underlying expertise compared the skill level of novices and experts, which gives a snapshot of two endpoints on the skill level continuum. So far, a growing number of investigations rather test subjects at different occasions to assess the gain of expertise throughout training period, hence the “novice state” can be compared with the “expert state,” or additionally in between (Guida et al., 2012). Using both approaches enables to infer the neural reorganization that contributes to reach the highest level of performance, which is impacted and reinforced through the consolidation processes (Diekelmann et al., 2011). This set of processes takes place automatically, without awareness, and allows the conversion of the initial unstable memory representation into a more stable and effective form, available for continued reactivation and recall over extended periods of time (Stickgold and Walker, 2007). Interestingly, processes of consolidation over time can also facilitate behavior, often through offline memory reorganization (Stickgold and Walker, 2005). Indeed, continued plasticity over time is crucial whenever newly acquired information are integrated with old memories (Abraham and Robins, 2005). Given this consolidation effect, some investigations include follow-up tests (for example, Draganski et al., 2004; Scholz et al., 2009). For all of these memory processes, sleep has been shown to play a critical role in meeting the demands of the organism (Walker and Stickgold, 2010; Rasch and Born, 2013). At the functional level, the consolidation refers to processes in which reverberating activity in newly encoded representations stimulate a redistribution of the neuronal representations to other neuronal circuitries for long-term storage (Dudai, 2004).

On the basis of several thousand years of education, along with more recent laboratory research on learning and skill acquisition, a number of conditions for optimal learning and improvement of performance have been uncovered. The most common condition for optimal learning and improvement of performance concerns the extended periods of deliberate practice (Ericsson et al., 1993). Four hours of physical/mental deliberate practice daily for approximately 10 years purportedly leads, for example, to expertise in chess, music composition, art, sport, and science (the 10-year or 10,000-h rule; Ericsson, 2008). One of the major interests for modern neuroscience is to investigate the plastic changes that occur in brain structures when people participate in such intensive motor and/or mental training. Nowadays, skill acquisition and training in various domains, from motor function to higher order cognitive skills, were shown to elicit substantial changes in brain anatomy (Jäncke, 2009). Previous studies including cross-sectional and longitudinal designs showed that skill acquisition and practice can induce changes in the functional properties of specific brain regions involved in the corresponding task (Buschkuehl et al., 2012; Guida et al., 2012). Recent investigations further demonstrated that not only brain activity, but also gray and white matter structures change as a consequence of skill learning (Draganski et al., 2004; Scholz et al., 2009). For instance, recent neuroimaging studies focusing on the effects of motor or mental (meditation) training, lasting several weeks in previously untrained participants, showed plastic changes in specific white matter regions (Scholz et al., 2009; Tang et al., 2012). Overall, technological and methodological advances in neuroimaging and non-invasive human brain stimulation provided insights into the neuroplastic mechanisms that underlie skill expertise. More generally, cross-sectional paradigms, where highly skilled participants were compared with less-skilled persons, showed a strong link between skill acquisition and neuronal plasticity at both cortical and subcortical levels over time, engaging different spatially distributed and interconnected brain regions.

A fundamental ability of the human brain is to form and retrieve memories enabling the individual to adapt its behavior to the demands of an ever-changing environment, and appropriately select and improve the behaviors of its given repertoire. The distinction between expertise based on declarative (*knowing that*) and procedural knowledge (*knowing how*) is directly related to the real-world domains of expertise. Some domains are mostly characterized by one type of knowledge, although experts may have both and often need to rely on one type of knowledge rather than the other (Dror, 2011). Practically, the most appropriate type of expert knowledge depends on the situational demands and cognitive mechanisms involved in operationalizing this knowledge (e.g., Beilock et al., 2004; Dror, 2011). Such kind of adaptation is a reliable marker of expertise. Interestingly, it has been shown that there are both costs and benefits to expertise, so that the inflexibility of experts might make them unable to adapt to new task demands, at least in some occasions (Bilalic et al., 2008a,b) Typically, experts may fail to develop successful patterns of thought by using a less familiar, albeit more appropriate solution, when available. Bilalic et al. (2008a,b, 2010a), however, demonstrated that individuals with the highest degree of expertise remained more flexible and more likely to find the optimal solution by memory retrieval and/or by search.

The primary aim of the present paper is to review studies showing how learning and experience induce structural and functional brain plasticity that supports expertise, following a motor to mental gradient of skill (**Figure 1**). We not only consider the structural and functional organization that the brain shapes along the course of gain in expertise, but also the brain’s potential to reorganize its functional organization by modifying its structure in response to practice. Meeting this challenge, this review integrates available results from several neuroimaging approaches in human populations and animal models. For the first time, we address these issues in exploring three domains of expertise which are usually disconnected (motor skill, mental simulation of movement, and meditation). We first report and underline separately the neuroanatomic correlates of expertise following a motor to mental gradient, and then attempt to identify and link the common patterns of changes in the brain subserving expert-level of performance within each domain. Practically, using such a transversal approach, this review aims at providing new insights about how brain plasticity occurs and supports the expert level of performance.

**FIGURE 1:**
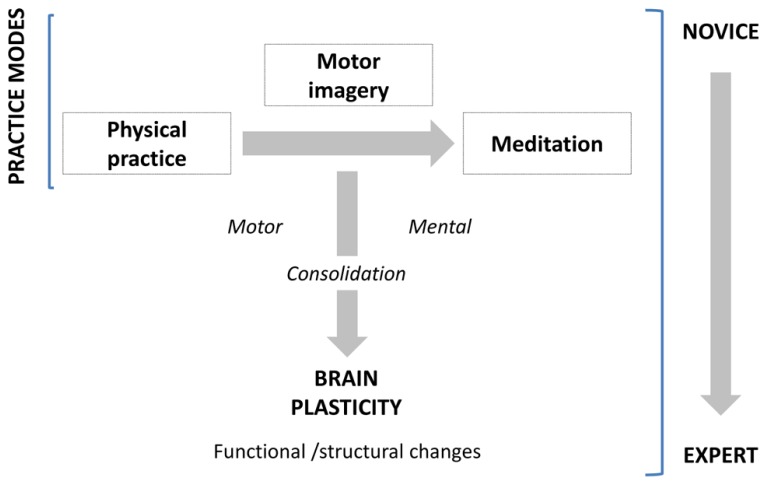
**Practice modes and consolidation processes leading to expertise**.

## WHEN MOTOR SKILLS EXPERTISE SUBSERVES BRAIN OPTIMIZATION

Recent years have seen a significant growing of knowledge on the neuroplasticity that underpins motor skill learning from its acquisition to the expert level of performance (for review, see Patel et al., 2013). Basically, compared to novices, highly trained individuals exhibit a number of differences including a reduction in the variability of repeated movements (Milton et al., 2004), in muscle activation (Lay et al., 2002), and a decrease in the overall volume of brain activation together with a relative increased intensity of activation in specific brain regions necessary for the execution of the task (Jäncke et al., 2000; Munte et al., 2002; Lotze et al., 2003). In our attempt to understand such robust expertise-dependent differences, we first report in this section the main data interesting how motor skills are acquired and enhanced through the consolidation process. Then, we review recent findings showing changes in the pattern of brain activity as well as modifications in the neural structures associated with expertise.

### THE NEUROCOGNITIVE BASIS OF MOTOR SKILL LEARNING

Motor skill acquisition refers to the process by which a movement is performed effortlessly through repeated practice and interactions with the environment (Willingham, 1998). Accurate motor performance is essential to almost everything we do, from typing to driving, or playing sports. In cognitive psychology, theoretical descriptions of changes in skilled performance were shown to move from cognitive to automatic processing (Fitts, 1964). The key concept is the increasing automaticity: controlled processes are attention demanding, conscious and inefficient, whereas automatic processes are rapid, smooth, effortless, require little attentional capacity, and are difficult to be consciously disrupted (Shiffrin and Schneider, 1977). Two experimental paradigms are used to investigate the cognitive processes and the neural substrates mediating our capacity to learn behaviors: the first measures the incremental acquisition of movements into a well-executed behavior (motor sequence learning), whereas the second tests our capacity to compensate for environmental changes (motor adaptation). Doyon and Benali (2005) proposed an integrated view of the functional plasticity that such a motor memory trace undergoes in each case. This model suggests that depending upon the nature of the cognitive processes required during learning, both motor sequence and motor adaptation tasks recruit similar cerebral structures early in the learning phase, including the striatum, cerebellum, and motor cortical regions, in addition to prefrontal, parietal and limbic areas. A shift of motor representation from the associative to the sensorimotor striatal territory can be seen during sequence learning, whereas additional representation of the skill can be observed in the cerebellar nuclei after practice on motor adaptation tasks. When consolidation has occurred, the participant has achieved asymptotic performance. However, the neural representation of a new motor skill is believed to be distributed in a network of structures involving the cortico-striatal and/or cortico-cerebellar circuits depending on the type of skill acquired. At this stage, the model suggests that motor adaptation rather involves the cerebellum for the retention and future executions of the acquired skill. By contrast, a reverse pattern of functional plasticity occurs in motor sequence learning, where the cerebellum is no longer essential, and the consolidation of the skill involves representational changes in the striatum and associated motor cortical regions. New insights into the neuroplastic mechanisms that underlie motor skill learning corroborate that skill acquisition is subserved by multiple mechanisms that operate across different temporal scales (Dayan and Cohen, 2011).

Practically, it is not automaticity *per se* that is indicative of high proficiency, but rather the level of skill at which automaticity is attained. Although the border between the automaticity and the expertise concepts beg for clarification, one may consider that most people fail to develop beyond a hobbyist level of performance as they settle into automaticity at a given level of skill that they find enjoyable, rather than continuing to improve skills (Ericsson, 2007). Hence, automaticity is more a false ceiling than a measure of excellence. Previous studies typically defined skill acquisition in terms of reduction in the speed of movement execution or reaction times, increase in accuracy, or decrease in movement variability. Yet, such measurements are often interdependent, in that faster movements can be performed at the cost of reduced accuracy and vice versa, a phenomenon which has been often referred to the speed-accuracy trade-off (Fitts, 1954). One solution to this issue is through assessment of changes in speed-accuracy trade-off functions, i.e., to defy the speed–accuracy tradeoff for a given task (Krakauer, 2009; Krakauer and Mazzoni, 2011). In other words, a skilled tennis player can serve both faster and more accurately than a novice. Thus, sporting skill at the level of motor execution can be considered as acquiring a new speed–accuracy trade-off relationships for each sub-task of the motor sequence.

### FUNCTIONAL PLASTICITY CHARACTERIZING NOVICES AND EXPERTS

The existing longitudinal studies in healthy participants attached great importance to controlled practice situations by keeping training parameters as much constant as possible (e.g., training duration per day, overall training duration, training schedule, strategies, etc.). Movement automatization reflects high level of motor skill performance and has been associated with increased activation in the primary motor cortex (M1), primary somatosensory cortex, supplementary motor area (SMA), and putamen, as well as decreased activation in the lobule VI of the cerebellum (Floyer-Lea and Matthews, 2005; Lehericy et al., 2005). Thus, training-related automaticity decreases in the fronto-parietal and dorsal attention networks, hence suggesting that progress from acquisition to automatization stages of motor skill learning is characterized by concomitant reduced demands on externally focused attention and executive function (Kelly and Garavan, 2005). Such pattern of activation is particularly elicited for motor sequence learning, but one would hypothesize that forms of motor and visuomotor learning which are more cognitive or associative in nature (Parsons et al., 2005) may recruit slightly different cerebral networks undergoing other patterns of plasticity with learning. For example, the preparatory period has been most extensively studied in athletes where it is called the “pre-shot routine.” Consistency and reproducibility of pre-shot routines are suggested to be among the most important differences that distinguish experts from novices in sport such as Golf (Feltz and Landers, 1983), compared to some motor skills such as goal kicking in rugby (Jackson, 2003) where no association between temporal consistency of the pre-shot routine and performance has been observed. In a recent study, Milton et al. (2007) found different functional activations during the pre-shot routine in expert and novice golfers. Especially, the posterior cingulate cortex, the amygdala–forebrain complex, and the basal ganglia were active only in novices, whereas experts yielded activation primarily in the superior parietal lobule, the dorsal lateral premotor area, and the occipital cortex. These results suggest that the disparity between the quality of the performance of novice and expert golfers lies at the level of the functional organization of neural networks during motor planning. More generally, Patel et al. (2013) demonstrated that spatially distributed cortical networks and subcortical striatal regions may serve as neural markers of practice interventions.

The expertise stage of motor skill learning, both in humans and animals, consistently reported either increased or decreased M1 activation depending on both the time interval and task complexity. On one hand, performing an explicit sequence of finger movements over several weeks showed a progressive increase of activity in M1 (Karni, 1995; Hlustik et al., 2004; Floyer-Lea and Matthews, 2005), hence reflecting recruitment of additional M1 units into the local network that represents the acquired sequence of movements (Ungerleider et al., 2002). Learning a motor sequence over several days was also accompanied by an increase in the size of motor maps and cortico-motoneuronal excitability of the body parts involved in the task (Pascual-Leone et al., 1995). Such plastic changes in M1 function linked with slow stage of motor skill learning are well established in animal models as well. For example, functional reorganization of movement representations in M1 has been documented in squirrel monkeys (Nudo and Milliken, 1996) and rodents (Kleim et al., 2004). It was found that an expansion in movement representations with training, detectable only after substantial practice periods, paralleled behavioral gains. Such findings challenged theories of neural efficiency proposing that optimized neural processing is associated with reduced activity in M1 (Jäncke et al., 2000; Krings et al., 2000; Haslinger et al., 2004). Especially, an increased activity of the motor network was reported, including M1, cerebellum, premotor cortex, basal ganglia, pre-SMA, and SMA in the initial acquisition of motor skills, with significant attenuation of activity following consolidation of the motor skill (Steele and Penhune, 2010). Recently, Picard et al. (2013) examined the consequence of practice-dependent motor learning on the metabolic and neural activity in M1 of monkeys who had extensive training (~1–6 years) on sequential movement tasks. They found that practicing a skilled movement and the development of expertise lead to lower M1 metabolic activity, without a concomitant reduction in neuron activity. In other term, they showed that less synaptic activity was required to generate a given amount of neuronal activity. The authors suggested that this gain in M1 efficiency might result from a number of factors such as more effective synapses, greater synchrony in inputs and more finely tuned inputs. They concluded that low activation in M1 elicited during extended practice might be a reflection of plastic mechanisms involved in the development of expertise. Although there is no clear consensus about M1 activation during the gain of expertise, it is more likely that time interval might be one of the main factors that play a role in brain modifications. This issue has been partially raised by Hotermans et al. (2008) who investigated the role of M1 in the different phases of motor memory consolidation by applying repetitive transcranial magnetic stimulation (rTMS) over M1 just after a training session involving a sequential finger-tapping task. They showed that interfering with M1 attenuated the early post-training performance without any detrimental consequences on the long-term behavioral improvement tested 4 or 24 h after. These results support that M1 is causally involved early after the acquisition of a new motor-skill but is no longer mandatory following a consolidation-period. Overall, increased activity in M1 might correspond to the integration of the new sensory information during the short period of training of a new skill, but when the new learned skill becomes automatic, M1 activation is less important. **Table 1** summarizes the human studies reviewed in this section with the brain areas showing early–late and expert-related functional plasticity.

**Table 1:** Functional plasticity through longitudinal and cross-sectional approaches: focus on the motor network.

Study	Task/method	Design	Main outcome
**Longitudinal**
*Normal adults*
Floyer-Lea and Matthews (2005)	Tracking sequential task/fMRI	Single training session vs. 15 min/session 5 weekdays/3 weeks	Early: ↘ activity DLPC, anterior cingulate, posterior parietal, M1 and cerebellar cortex
			Long-term: ↗ activity left S1, M1 and right putamen
Steele and Penhune (2010)	FTT/fMRI	5 weekdays	Early: ↗ cerebellum, PMC, basal ganglia, pre-SMA and SMA
			Long-term: ↘ in these regions
Karni (1995)	FTT/fMRI	10–20 min daily session/3 weeks	Greater activation in M1 was significantly compared to the extent of activation evoked by an untrained sequence
Hlustik et al. (2004)	FTT/fMRI	15 min daily session/3 weeks	Gradual expansion of M1 and S1 correlating with performance
Hotermans et al. (2008)	FTT/rTMS	rTMS immediately before testing at 30 min, 4 or 24 h after practice	Early acquisition M1 supports performance, but is no longer mandatory following consolidation
Parsons et al. (2005)	FTT/fMRI	5 weekdays	Early: ↗ activation in motor areas and ant/post CMA
			Long-term: ↘ activation cerebellum, motor areas, striatum, fronto-parietal cortices
Pascual-Leone et al. (1995)	FTT/TMS	2 h daily training vs. no-training control 5 days	Following training: hand motor areas enlarged while activation ↘
**Cross-sectional**
*Pianists vs. novices*
Haslinger et al. (2004)	FTT/fMRI		Pianists recruited an extensive motor network but with a lesser degree of activation than novices
Jäncke et al. (2000)	FTT/fMRI		Less activation in M1, SMA, pre-SMA, and CMA in pianists
Krings et al. (2000)	FTT/fMRI		Pianists showed less activation in M1, SMA, and PMC

*FTT, finger tapping task; rTMS, repetitive transcranial magnetic stimulation; fMRI, functional magnetic resonance imagery; ↗, increase; ↘, decrease; M1, primary motor cortex; S1, primary somatosensory cortex; DLPC, dorsolateral prefrontal cortex; SMA, supplementary motor area; pre-SMA, pre-supplementary motor area; PMC, premotor cortex; CMA, cingulate motor areas.*

An alternative explanation for the modulation of M1 activity has been suggested by Landau and D’Esposito (2006) who reported that optimized motor system is capable of greater flexibility and adaptability, depending on the nature of the task demands. Using a complex sequential finger task, they found increased motor activations in pianists than non-pianists, and further argued that the former may yield decreased activation when they were less challenged. Further research is therefore needed to determine the modulation of M1 activation in experts and novices using task characteristics that vary across several degrees of difficulty either early or late during the learning process.

### EXPERIENCE-DEPENDENT STRUCTURAL CHANGES IN THE HUMAN BRAIN

Besides to the functional reorganization of the brain motor networks, current neuroimaging studies suggest that physical practice is also reflected in macroscopic changes in motor-related structures (Draganski and May, 2008). Practically, higher gray matter volume in auditory, sensorimotor, and premotor cortex, as well as in the cerebellum, was reported in musicians compared to non-musicians (Gaser and Schlaug, 2003). Related findings have been reported in many other specific tasks, including typing (Cannonieri et al., 2007), basketball (Park et al., 2009, 2011), or golf performance (Jäncke et al., 2009; Bezzola et al., 2011). Although these findings tend to follow the law “more skill, more gray matter,” there is new evidence that training and ensuing expertise may induce local decrease of cortical volume (Hänggi et al., 2010; Granert et al., 2011). Controversial findings were also reported on training-related plasticity in white matter microstructure in healthy adults, although fewer studies dealing with this issue have been published.

On the one side, numerous investigations reported a strong association between specialized skill and structural changes in particular brain structures (Maguire et al., 2000; Hutchinson et al., 2003; Mechelli et al., 2004; Park et al., 2009). In a longitudinal study, Draganski et al. (2004) used a complex visuo-motor juggling task where perception and anticipation of moving targets determined the planning of the subsequent motor action. Young volunteers were scanned before and after 3 months of a daily training period. The results showed a transient bilateral expansion in gray matter in the mid-temporal area (hMT/V5) and the left inferior parietal sulcus. The authors concluded that juggling, and consequently the perception and spatial anticipation of moving objects, is a stronger stimulus for structural plasticity in visual areas than in motor areas. Similarly, cross-sectional neuroimaging studies showed experience-dependent structural plasticity in the cerebellum following training of complex motor skills (Park et al., 2009; Scholz et al., 2009). For example, Park et al. (2012) found greater right- than left cerebellar volume asymmetry and relatively larger volumes of right hemisphere and vermis lobules VI–VII (declive, folium, and tuber) in short-track speed skating players compared to matched controls. This finding suggests that the specialized abilities of balance and coordination are associated with structural plasticity of the right hemisphere of cerebellum and vermis VI–VII, these regions playing a critical role in balance and coordination. Cannonieri et al. (2007) further found that the volume of brain areas corresponding to the motor skill increased proportionally to the duration of the training period. Together, these findings demonstrate that practice modulates brain anatomy specifically associated with practice demands, albeit opposite structural neural correlates reflecting stepwise increases in expertise have also been found. For instance, Hänggi et al., (2010) found differences in structural characteristics within the sensorimotor neural network between professional women ballet dancers and novices. Especially, they reported decreased gray matter volumes in the left premotor cortex, the SMA, the putamen, and the superior frontal gyrus anterior to the premotor cortex. More recently, James et al. (2014) reported changes in gray matter as a function of musical training intensity in three groups of young adults (non-musicians, amateurs, and professionals). Surprisingly, they observed a progressive increase of gray matter density with respect to the level of expertise in several regions involved in higher-order cognitive processing (e.g., right fusiform gyrus activated for visual pattern recognition), whereas an opposite pattern of results were found in sensorimotor areas. To summarize, the type of density changes (increase or decrease) and the localization of structural plasticity in gray matter may be related to different factors such as the nature of the motor-skill, duration and stage of practice.

Nowadays, investigations are designed to determine the time scales of gray matter changes from novices to experts (Taubert et al., 2010; Dayan and Cohen, 2011). So far, the process of gray matter adaptation has been observed as early as following 7 days of practice (Driemeyer et al., 2008) and as late as after 6 weeks (Scholz et al., 2009), hence demonstrating a relatively fast and durable structural gray matter plasticity. Although interpretation of such striking results are premature, it has been proposed that processes occurring both at the synapse level and larger scales (e.g., glial hypertrophy), may play a contributory role (Draganski and May, 2008).

Recent longitudinal neuroimaging studies further focused on the effects of motor training lasting several days or weeks in previously untrained participants, and showed specific structural plasticity in white matter regions (Scholz et al., 2009; Taubert et al., 2010). Scholz et al. (2009) reported experience-induced changes in white matter architecture following a short period of practice. Practically, it was found that 6 weeks of juggling practice protracted an increased fractional anisotropy in a region of white matter underlying the intraparietal sulcus. Interestingly, Della-Maggiore et al. (2009) further demonstrated that the speed in a visuomotor adaptation task might be partially determined by the variation of fractional anisotropy in the posterior cerebellum and superior cerebellar peduncle. Together, these findings show that rates of motor skill practice might correlate with higher values of fractional anisotropy at a local level triggered by the nature of the motor task. Cross-sectional studies, primarily in highly trained musicians, also examined white matter correlates of skilled behavior (Schmithorst and Wilke, 2002; Bengtsson et al., 2005; Han et al., 2009). Bengtsson et al. (2005) found a correlation between fractional anisotropy in the posterior limb of the internal capsule, which contains descending corticospinal fibers from the primary sensorimotor and premotor cortices, with number of practice hours during childhood in skilled musicians. These results demonstrate that training during a critical developmental period may induce local structural plasticity. Rüber et al. (2013) further reported higher fractional anisotropy values in musicians than non-musicians in descending motor tracts, with differences when practice was a fine finger unimanual or bimanual motor skill. The matter tracts were modified to reflect specific motor demands, unimanual motor skill primarily eliciting structural remodeling of right hemispheric tracts, and bimanual motor skill leading to bilateral structural tract remodeling. Accordingly, Roberts et al. (2012) compared the behavior and brain structure of healthy controls with a group of karate black belts, an expert group who are able to perform rapid, complex movements that require years of training. As expected, experts were more able than novices to coordinate the timing of inter-segmental joint velocities, and data revealed significant group differences in the microstructure of white matter in the superior cerebellar peduncles and M1, these brain regions participating to the voluntary control of the movement. Overall, as experts demonstrate optimal behavior on specific tasks, differences in white matter structure relative to novices might reflect a “fine-tuning” of the connectivity between specific brain regions. Nevertheless, contrasting patterns of results shows lower white matter values in experts than in non-experts. In order to disentangle the unresolved question of whether sensorimotor training leads to increased or decreased white matter density, Imfeld et al. (2009) investigated the corticospinal tract of musicians using different methods of analysis (fiber tractography, voxelwise analysis, region of interest analysis, and detailed slicewise analysis of diffusion parameters). Data clearly demonstrated that sensorimotor training leads to decreased white matter density. This finding is supported by Hänggi et al. (2010) who found reduced white matter volume in dancers vs. non-dancers. Further investigations are required to understand the cellular mechanisms underlying learning-dependent changes in white matter microstructure.

## THE NEURAL CORRELATES OF MOTOR IMAGERY

Across a motor to mental gradient of skill learning, there is now compelling evidence that motor imagery contributes to enhance motor skill learning and motor performance (for reviews, see Feltz and Landers, 1983; Driskell et al., 1994; Guillot and Collet, 2008; Schuster et al., 2011). Motor imagery is a dynamic state during which one simulates an action mentally without any concomitant body movement. Since the last three decades, the advent of functional brain mapping studies has allowed researchers to investigate the neural correlates of motor imagery and to understand in greater details the neural underpinnings of expertise in imagery.

### THE EFFECT OF EXPERTISE LEVEL

In previous sections, we reviewed a handful of experimental studies providing clear evidence that individuals differ in their ability to perform voluntary motor acts, and that advanced motor learning is associated with functional brain plasticity (for reviews, see Doyon and Benali, 2005; Doyon, 2008; Johansson, 2011). Practically, neuroimaging studies showed that the neural networks activated by the execution of the motor task differed as a function of the individual expertise level. Although there are few studies looking at this issue in motor imagery, similar observations were reported in the neural activations between novices and experts. For instance, by comparing the neural substrate of judgment processing in amateurs and professionals “Go players,” Ouchi et al. (2005) found that in the checkmate-decision problems, the precuneus and cerebellum were activated in the professionals, while the premotor and parieto-occipital cortices were extensively activated in the amateurs. Their results support that the precuneus and the cerebellum play a crucial role in processing of accurate judgment by visual imagery. Likewise, brain imaging studies investigating object and pattern identification, such as pieces on chess and their respective prospective functions (e.g., imagining moving a piece to capture another), revealed that expertise modulates the activity of several regions. Accordingly, Bilalic et al. (2010a,b, 2011) showed that chess-specific object recognition was accompanied by bilateral activation of the occipito-temporal junction, while chess-specific pattern recognition was related to bilateral activations in the middle part of the collateral sulci. More generally, Bilalic et al. (2012) provided evidence that experts not only engage the same regions as novices, but also recruit additional regions including bilateral activation of the retrosplenial cortex, the collateral sulcus, and the temporo-parietal region (see also Rennig et al., 2013), hence suggesting that the pattern of activation moves from frontal parts at the beginning of the process to posterior parts responsible for retrieval of domain specific knowledge around the final expertise stage.

Several other studies were designed to investigate the neural networks mediating the phenomenological experience of imagining music (Halpern and Zatorre, 1999; Halpern et al., 2004; Zatorre et al., 2007; for reviews, see Zatorre and Halpern, 2005; Lotze, 2013). A nice study by Lotze et al. (2003) compared the patterns of brain activation during auditory imagery in experienced and novice musicians, with the formers reporting high vividness and frequent use of imagery. Interestingly, they showed that experienced musicians overall recruited fewer cerebral areas, while amateurs manifested a widely distributed activation map. In the professional group, however, more activation was observed in regions assuming motor functions including the SMA, the superior premotor cortex, and the cerebellum, as well as in the superior parietal lobule. By means of magnetoencephalography, Herholz et al. (2008) also compared musicians and non-musicians during imagery of a musical task. An early pre-attentive brain response (imagery mismatch negative response) to unexpected continuations of imagined melodies was observed only in musicians, hence reflecting the neuroplasticity due to intense training for music processing.

Similar experiments were conducted in the field of motor imagery *per se* by comparing the neural networks mediating the imagery experience in novices and elite athletes (e.g., Milton et al., 2008). Especially, Ross et al. (2003) compared the brain activations of six participants during motor imagery of a golf swing. They found an inverse relationship between brain activity and skill level, i.e., decreased activations occurred with increased golf skill level, especially in the SMA and cerebellum. Also, imagined golf swing elicited little activation of basal ganglia and cingulate gyri across all skill levels (see also Milton et al., 2007). Wei and Luo (2010) compared the pattern of cerebral activations in professional divers and novices during imagery of both professional (diving task) and simple (basic gymnastics task) motor skills. Elite athletes yielded greater activation in the parahippocampus during imagery of professional skills and a more focused activity of the prefrontal regions in both tasks. In a comparable study in archers, Chang et al. (2011) reported peaks of activation in premotor and SMA, in the inferior frontal region, as well as in basal ganglia and cerebellum, in novices. In contrast, elite archers involved predominantly the SMA, hence confirming a more focused pattern of activity following intense training. The between-group analysis revealed that novices exhibited significantly higher activation in M1, premotor area, inferior parietal cortex, basal ganglia, and cerebellum.

**FIGURE 2:**
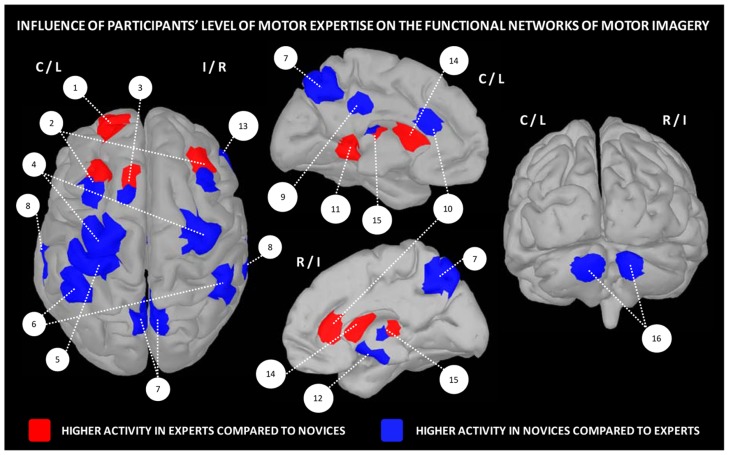
**Influence of the level of motor expertise on the functional networks underlying motor imagery.** 1 – frontal cortex (BA10), 2 – pre-motor cortex, 3 – SMA, 4 – M1, 5 – primary somatosensory cortex, 6 – inferior parietal cortex, 7 – precuneus, 8 – superior temporal lobe, 9 – posterior cingulate cortex, 10 – anterior cingulate cortex, 11 – parahippocampal gyrus (BA36), 12 – hippocampus, 13 – insula/inferior frontal cortex, 14 – striatum, 15 – putamen, 16 – cerebellum (lobules IV/V region). Data were extracted from Lafleur et al. (2002), Lacourse et al. (2005), Milton et al. (2007), Olsson et al. (2008), Wei and Luo (2010), Chang et al. (2011), Baeck et al. (2012), Bezzola et al. (2012).

Overall, these data strongly support the existence of distinct neural mechanisms of motor expertise during imagery, as a function of the individual skill level. Interestingly, dynamic changes resulting from intense practice of a given motor task tend to support a reduction of the general cortical activation during motor imagery, with a more refined and circumscribed pattern of activity in trained participants (**Figure 2**).

**FIGURE 3:**
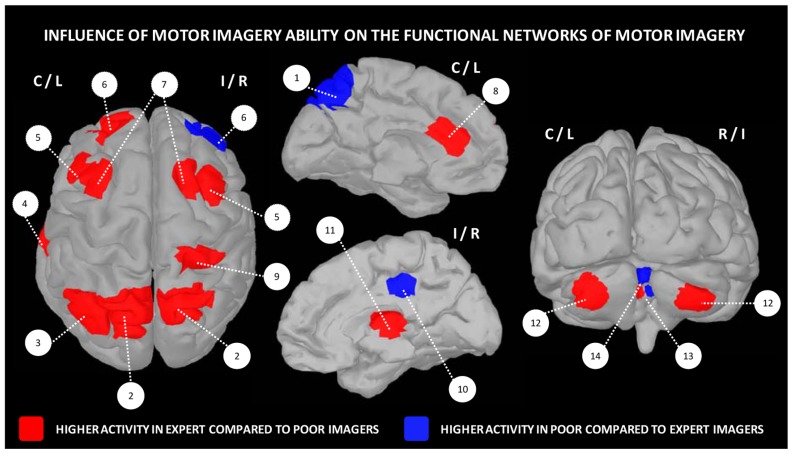
**Influence of motor imagery ability on the functional networks underlying motor imagery.** 1 – precuneus, 2 – superior parietal cortex, 3 – inferior parietal cortex, 4 – fusiform gyrus, 5 – lateral premotor cortex, 6 – frontal cortex, 7 – dorsal premotor cortex, 8 – anterior cingulate cortex, 9 – post-central gyrus, 10 – posterior cingulate cortex, 11 – thalamus, 12 – cerebellar cortex, 13 – cerebellar lobule IV, 14 – cerebellar lobules IV/V. Data were extracted from Guillot et al. (2008), Lorey et al. (2009), Hasenkamp et al. (2012).

Apart differences in terms of motor expertise, there is now ample evidence that the expertise in the use of imagery, which commonly refers to the individual imagery ability, also widely varies across individuals. It is therefore possible distinguishing good from poor imagers. Yet, very few experimental studies investigated the functional neuroanatomical correlates of imagery ability/expertise (**Figure 3**). Guillot et al. (2008) compared the pattern of cerebral activations in 13 skilled and 15 unskilled imagers, during both physical execution and imagery of a finger movement sequence. As expected, both groups manifested similar peaks of activation in many cerebral regions (inferior and superior parietal lobules, as well as motor-related regions including the lateral and medial premotor cortex, the cerebellum and putamen). Inter-group comparisons revealed, however, that good imagers activated more the parietal and ventrolateral premotor regions, known as playing a crucial role in the formation of the mental images. In contrast, poor imagers recruited the cerebellum, the orbito-frontal and posterior cingulate cortices. With reference to the motor sequence learning literature (Doyon and Ungerleider, 2002; Doyon and Benali, 2005), these findings strongly support that the neural networks mediating expertise in motor imagery are not identical in high and low-skilled individuals. Findings also suggested that compared to poor imagers, good imagers have a more efficient recruitment of movement engrams. In a recent study, van der Meulen et al. (2014) investigated the effect of imagery ability/expertise on the neural correlates of gait control. They confirmed that both good and poor imagers groups activated a network of similar brain activations. Good imagers, however, showed greater activity in the motor-related areas including the left M1, right thalamus and bilateral cerebellum, as well as the left prefrontal cortex contributing to higher order gait control. A greater activation was also found in the right SMA. Differences in the experimental designs as well as the criteria for determining the individual imagery ability may explain the differences between the two latter studies in terms of brain activations. Despite this, these studies provide a better understanding of the neural networks underlying imagery ability/expertise and highlight the importance of assessing the ability of participants to generate accurate mental images in order to adjust and individualize the content of mental practice programs.

### BRAIN PLASTICITY FOLLOWING MOTOR IMAGERY PRACTICE

We provided evidence that the neural networks underlying motor imagery in novices and elite athletes are not totally overlapping but selectively depend upon the individual level of motor expertise and imagery ability. Based on this assumption, one may postulate that the pattern of cerebral activation recorded during imagery in poor imagers and/or novices might improve and evolve close to that observed in good imagers and/or experts with practice. This might suggest that the expected changes in subcortical and cortical activations during motor imagery would reflect those elicited by the process of actual motor learning. In a pioneering series of experimental studies, Lacourse et al. (2004, 2005) reported functional cerebral and cerebellar sensorimotor plasticity following either physical or motor imagery practice. In line with such hypothesis, Lafleur et al. (2002) and Jackson et al. (2003) demonstrated that the functional plasticity occurring during the incremental acquisition of a motor sequence was also reflected during motor imagery. In other words, the patterns of dynamic changes in cerebral activity were significantly different when comparing both early and more advanced learning phases of imagined sequential foot movements. In particular, Lafleur et al. (2002) observed significant differences medially in the rostral portion of the anterior cingulate and orbito-frontal cortices, as well as in the striatum, bilaterally. Jackson et al. (2003) also explored the functional cerebral reorganization following motor imagery learning of a similar task. They confirmed the robustness of Lafleur et al.’s (2002) findings by a regression analysis, which showed a positive correlation between the increase in cerebral blood flow within the right medial orbito-frontal cortex and the individual performance enhancement. Recently, Baeck et al. (2012) confirmed the neuroplasticity elicited by motor imagery practice, while Bezzola et al. (2012) demonstrated that performing intensive physical training influences subsequent motor imagery of the corresponding task.

### CONCLUSION

The results reviewed in this section support the existence of distinct neural mechanisms for expertise in imagery. Accordingly, the neural networks mediating the imagery experience in individuals with poor imagery ability are not totally similar to those observed in high imagers. As well, comparisons between expert athletes and novices demonstrated different patterns of brain activation during motor imagery of the corresponding task. At this stage, one cannot totally rule out that the modulation in brain activity might also arise from the visual familiarity by experts of the movement to be imagined. Accordingly, higher activation might thus arise by the fact that it’s easier to imagine an action either because experts can perform the action or because they see/experience this action extensively in daily life. Such comparisons between expert and non-expert athletes, or between good and poor imagers, thus suffer from a major limit related to the familiarity with the movement to imagine, regardless the motor experience *per se*.

An interesting result supporting the functional equivalence between motor imagery and motor performance is that the functional plasticity that occurs during mental practice was found to closely mimic that observed after physical practice of the same motor skill. Imagery training may thus result in dynamic plastic changes so that the neural networks mediating imagery practice in poor imagers become closer to those observed in good imagers. Along these lines, real-time image analyses in functional magnetic resonance imaging (fMRI) studies may provide to participants some objective information related to the vividness of their imagery content. This method might be particularly useful during the imagery learning process, with regards to the potential modification of the mental images, when the pattern of activation during mental simulation is not the one expected. In other words, the participant may directly modulate his/her level of motor imagery expertise.

A significant illustration of the strength of this methodology in the field of motor imagery was offered by de Charms et al. (2004) during imagery of a manual action task. In this study, participants received feedback about the activation level in the somatomotor cortex with a simple virtual reality interface. The results showed that they enhanced the level of activation driven by motor imagery in the somatomotor cortex through the course of training. Moreover, the activation of this region after imagery training was as robust as that recorded during actual practice. Yoo et al. (2008) later showed that real time fMRI might help individuals to learn how to increase region-specific cortical activity associated with a motor imagery task. Practically, the level of increased activation in motor areas was consolidated after the 2-week self-practice period. More recently, Xie et al. (2011) supported the effectiveness of delivering neurofeedback during motor imagery using real-time fMRI, and further provided evidence that the SMA was controllable by participants. These data strongly support that real-time fMRI is a valuable technique to investigate whether participants are able to use a cognitive strategy to control a target brain region in real-time, and that motor imagery can reflect plastic changes of neural correlates associated with intensive training (Baeck et al., 2012).

## MENTAL TRAINING: MEDITATION AS A PARADIGMATIC EXAMPLE

In this section, we discuss recent findings on the cognitive processes and the neural underpinning supporting a pure mental task, i.e., meditation. First, we briefly introduce the concept of meditation and give some definition broadly diffused in the neuroscientific community, then we review findings on the cognitive enhancement linked to different levels of meditative expertise. Finally, we expose recent findings on the brain structural and functional changes associated with long-term meditation practice and the neuronal underpinning of meditation. In the last two sections, we mainly take into account data from functional and structural neuroimaging studies without considering results from the large body of researches using electroencephalography (we address readers to some recent extensive reviews of this field, e.g., Cahn and Polich, 2006; Travis and Shear, 2010).

### MEDITATION: BASIC CONCEPTS

Meditation is commonly used in the literature as an umbrella term that covers different practices ranging from yoga, tai-chi, transcendental meditation and different techniques derived from the Buddhist tradition. Here we will mainly focus on the latter, since they have received great attention in the last years, also due to their clinical application (Rubia, 2009), and since they focus on the training of well-defined cognitive processes. Indeed, a common feature of these practices is the voluntary control of the attentional focus. A distinction between focused attention (FA) and open monitoring (OM) techniques has been proposed (Lutz et al., 2008). FA practices are based on the concentration of attention on a particular external, corporal, or mental object while ignoring all irrelevant stimuli. At the opposite, OM techniques try to enlarge the attentional focus to all incoming sensations, emotions, and thoughts from moment to moment without focusing on any of them with a non-judgmental attitude (Lutz et al., 2008).

FA is thought to not only train sustained attention but also to develop three attentional skills: the monitoring and vigilance to distracting stimuli beyond the intended focus of attention, the disengagement of attention from distracting stimuli once the mind has wondered, and the redirection of FA on the intended object. OM meditation involves more monitoring processes of one’s own phenomenological experience and is thought to develop awareness and non-reactive meta-cognitive monitoring (Lutz et al., 2008; Slagter et al., 2011).

Expertise in other domains (e.g., sport, music) is defined as the achievement of better performance compared to novices in the particular activity that is trained. Following this definition it is not clear what could be an objective measure of expertise in meditation, since there are no clear assessments of “meditative performance,” and the quality of meditation is at best measured by subjective introspective reports. For this reason, the only consensual measurable parameter of expertise is the extent of practice reported in years or hours per week of practice. Nevertheless, an indirect quantitative measure of expertise could be the improvement of cognitive functions that are supposed to be trained during meditation. Thus, in the following section, we start presenting behavioral results that have highlighted cognitive improvements in expert meditators.

### COGNITIVE ENHANCEMENT INDUCED BY MEDITATION

Meditation training has been recently shown to improve different cognitive abilities. One of the most investigated domains, not surprisingly, is that of attentional improvement after either long- or short-term meditative practice.

Following the distinction of attentional processes proposed by Posner and Petersen (1990) in three subdivisions comprising alerting (the ability to reach and maintain a vigilance state), orienting (the capacity of focusing attention on a subset of stimuli) and conflict resolution or executive attention (the ability to resolve conflict or allocate limited resources between competing stimuli), different researchers have employed the Attentional Network Task (Fan et al., 2002) to investigate the impact of meditation on each subcomponent. Results on expert mindfulness meditators showed better performance in the orienting component and a trend to better executive attention (van den Hurk et al., 2010). These findings are consistent with other studies reporting enhanced sustained attention in expert meditators (Valentine and Sweet, 1999; Pagnoni and Cekic, 2007; Josefsson and Broberg, 2010).

In another study, Jha et al. (2007) compared performance on a similar task between a group of expert meditators, a group that followed a 5-week mindfulness training, and a control group. They further compared the performance between the three groups at baseline (T1) and after a 1-month meditative retreat for the meditators group, the 5-week training for the mindfulness group, and no treatment for the control group (T2). Their findings showed that at T1, experts had better performance in conflict monitoring compared to the other two groups, while at T2 the mindfulness group showed enhanced orienting and the retreat group performed better in the alerting component. In the same vein, Tang et al. (2007) reported that a short 5-days meditation training enhanced performance in conflict monitoring.

Other results in accordance with a benefit of meditation on the executive component of attention come from a study investigating the attentional blink effect. This effect consists on the fact that if two target stimuli (t1 and t2) are presented in rapid succession, normally the t2 is not detected (Raymond et al., 1992; Ward et al., 1996). Slagter et al. (2007) reported that after 3 months the intensive meditative practice the attentional blink was reduced as the result of reduced neurocognitive resources allocated to t1, as evidenced by a reduced electrophysiological brain potential (the P3b) normally associated with attentional resource allocation. These results, using a similar paradigm, were also replicated in a sample of more expert meditators (van Leeuwen et al., 2009).

Taken together, these findings suggest that the more affected component of the attentional network would be the executive one, even if some studies reported better performance on the orienting or the alerting component (van den Hurk et al., 2010). It should be noted, however, that participants in this study had a much longer meditation experience (mean 14.5 years) than in the others. This could suggest that the executive system is the first attentional component to benefit from meditation training and that longer practice is needed to achieve improvement in the other systems.

Beyond attentional performance, preliminary findings have shown that meditation could have a beneficial effect on other cognitive functions. For example, some studies reported better performance in participants assigned to a mindfulness meditation group on several executive functions such as verbal fluency (Wenk-Sormaz, 2005; Heeren et al., 2009; Zeidan et al., 2010), cognitive inhibition (Heeren et al., 2009) and working memory (Chambers et al., 2008; Zeidan et al., 2010; Mrazek et al., 2013).

Moreover, it has been shown that short-term meditation training can have a positive effect on autobiographical memory specificity in formerly depressed patients (Williams et al., 2000) and also in non-clinical population (Heeren et al., 2009), but these effects are largely mediated by enhanced performance in executive functions (Heeren et al., 2009).

### STATE-DEPENDENT FUNCTIONAL ACTIVATION DURING MEDITATION

In this section, we mainly focus on the ongoing brain activity during the practice of meditation and in particular on the evolution of this activity with the extent of practice. Before starting reviewing existing data, we want to underline that since meditation is a long-lasting state, in which different parallel cognitive processes coexist, that could be achieved with a variable lapse of time, it is a phenomenon that is difficultly suitable for current neuroimaging investigation. Indeed, the temporal course for entering the meditation state is unknown and variable across participants. One interesting solution has been recently applied by Hasenkamp et al. (2012). In their study, they asked participants who performed FA meditation to press a button as soon as they realize their attention was wandering. Using this stratagem the authors were able to dissociate different aspects of the meditative process: mind wandering, awareness of mind wandering, shift of attention and FA. Moreover, in trying to adapt meditation task to neuroimaging protocol, most of the previous studies used blocked designs with short meditation period (i.e., from 30 s to several minutes); this methodology may not reflect the complexity of meditative processes. Thus, results from functional neuroimaging studies on meditation should be taken with caution. Nevertheless, several activated regions were constantly reported across different studies and meditative techniques as pointed out by two recent meta-analyses (Sperduti et al., 2012; Tomasino et al., 2013).

Different studies have reported that meditation is supported by a large set of brain areas encompassing lateral and medial frontal regions, comprising the anterior cingulate cortex (ACC), parietal structures, the insula, and medial temporal structures such as the hippocampus and the parahippocampal formation, and the basal ganglia (Brefczynski-Lewis et al., 2007; Hölzel et al., 2007; Bærentsen et al., 2010; Engström et al., 2010; Manna et al., 2010; Wang et al., 2011; Sperduti et al., 2012; Tomasino et al., 2013). Frontal and parietal regions encompassing the ACC are thought to reflect cognitive control and attentional monitoring during meditation, while insula is known to be involved in interoceptive awareness. On the contrary, the role of hippocampus during meditation is still a matter of debate and could reflect memory consolidation, emotional regulation, or spontaneous thoughts monitoring (Engström et al., 2010; Sperduti et al., 2012).

Nevertheless, establishing a unitary neural correlate of the complex task of meditation is not without problems. Indeed, different studies have shown that the neural underpinning of meditation could differ depending on the specific meditative technique that is investigated and the expertise of the participants. For example, Manna et al. (2010) directly compared FA and OM meditation in the same participants, and reported that OM more strongly activated lateral prefrontal regions. In the same vein, Wang et al. (2011) showed that a breath-based meditation (defined by the authors as a non-FA technique), compared to mantra repetition (a form of FA meditation), activated to a greater extent limbic structures (hippocampus, parahippocampus, and amygdala), insula and lateral frontal areas, while mantra repetition was more associated with activations in the precentral gyrus, parietal cortex, and medial frontal gyrus. Moreover, the engagement of certain structures, above all frontal regions, could vary with the individual expertise. Indeed, Brefczynski-Lewis et al. (2007) reported an inverted U-shaped relation between frontal activity and meditators’ expertise. In other words, the more trained persons showed a less frontal activity, suggesting a disengagement of attentional control with training. These results are in line with a recent meta-analysis by Sperduti et al. (2012) showing that pooling together studies on expert meditators belonging to different meditative techniques did not report frontal activity, but common activation in the basal ganglia, medial prefrontal cortex, and parahippocampus. These data suggest that some brain regions, in particular those involved in cognitive control, are necessary at the initial and intermediary levels of expertise, while with practice meditation may be a highly automatic and effortless process (Lutz et al., 2008). Moreover, while the neurocognitive underpinnings of different meditative practices would differ in novices, it could eventually converge when expertise is attained, as suggested by Newberg and Iversen (2003, p. 283): “*Phenomenological analysis suggests that the end results of many practices of meditation are similar, although these results might be described using different characteristics depending on the culture and individual. Therefore, it seems reasonable that while the initial neurophysiological activation occurring during any given practice may differ, there should eventually be a convergence*.”

### TRAIT STABLE MODIFICATION OF BRAIN ORGANIZATION ASSOCIATED WITH LONG-TERM MEDITATION

One of the first studies investigating long-term effect of meditation on brain morphology is that of Lazar et al. (2005). The authors reported greater cortical thickness in meditators than controls in the prefrontal cortex, the insula, and the somatosensory cortex. Moreover, they found a positive correlation between cortical thickness and expertise in the occipito-temporal visual cortex. Increase in gray matter concentration in the insula was further confirmed by Hölzel et al. (2008), who additionally reported greater gray matter concentration in the right hippocampus and left inferior temporal gyrus. In this latter region, the amount of gray matter concentration positively correlated with that of meditation training. Hippocampal alteration in expert meditators has also been reported in several recent studies (Luders et al., 2009, 2013). Another structure that has been found to be altered in meditators is the putamen (Pagnoni and Cekic, 2007). Indeed, while in aging this region normally shows a decrease in gray matter concentration, this was not the case for a group of expert meditators. This result, together with findings suggesting a protective effect of long-term meditation on the cognitive decline in aging (Prakash et al., 2012), opens the interesting perspective of employing meditation as a neurocognitive training technique in elderly.

There are also some studies reporting white matter changes induced by meditation. Luders et al. (2011) observed widespread increased of fractional anisotropy, a measure of fiber tracks integrity through the brain in expert meditators compared to controls. Similar results have been reported in a recent study by Kang et al. (2013). Moreover, two interesting studies on novice meditators showed that only 11 h of meditation training increased fractional anisotropy in the corona radiata (Tang et al., 2010, 2012), a tract connecting the ACC with other cortical structures. Increased fractional anisotropy could be interpreted as an enhanced connectivity between large scale networks. Studies reporting altered resting state functional connectivity in meditators are compatible with this interpretation. Indeed, several studies reported both increased connectivity within the default mode network (Brewer et al., 2011; Jang et al., 2011) and the attentional network (Hasenkamp and Barsalou, 2012), and between these two networks (Brewer et al., 2011; Hasenkamp and Barsalou, 2012; Taylor et al., 2013).

These findings confirm that long-, but also short-term meditative practice, could lead to stable alterations at the structural level in gray and white matter, and in a functional rewiring of large scale networks. Regions that are active during meditation are the most affected by these structural changes, and in some cases, expertise has been reported to correlate with the magnitude of morphological changes.

### DISCUSSION

In the previous sections, we reviewed findings showing that meditation is subserved by a widespread network of brain regions involved in different processes such as cognitive control, attention, and interoceptive awareness. Moreover, the engagement of some of these structures seems dependent on the degree of expertise, with frontal regions being possibly involved at initial stages of practice, while at more advanced stages, when meditation practice eventually becomes “effortless,” basal ganglia seem to play a central role. The continuous training in meditation, possibly resulting on the repeated activation of this set of regions, produces long-term structural and functional changes at the local level, but also in long range brain connectivity.

At the behavioral level, meditation has been shown to produce not only an enhancement in attentional performance that is supposed to be directly trained during practice, but also to improve different cognitive processes, such as executive functions, working memory, and long-term autobiographical memory. These findings are in line with the proposal of Slagter et al. (2011) stating that meditation could promote “process-specific learning” that is a kind of learning which is not confined to the enhancement of performance in the trained task, but could transfer to other tasks and domains.

Since most of the evidence about the relationship between meditative practice and cognitive, structural, and functional brain alteration comes from cross-sectional studies, conclusion about the causal role should be taken with caution. Nevertheless, the correlations often reported between the degree of expertise and the amount of performance or neural modifications, together with some recent longitudinal studies on short-term meditative training, seem to corroborate this interpretation. Further efforts should be done to carry out longitudinal studies that could shed light on the progressive cognitive and brain reorganization induced by different levels of meditative expertise.

## GENERAL CONCLUSIONS

Technological and methodological advances in neuroimaging and non-invasive brain stimulation in humans, along with novel findings stemming from animal studies, provide new insights into the neuroplastic mechanisms underlying expert level of performance, and suggest that multiple mechanisms operate across different temporal scales from skill acquisition to expertise. In this review, we attempted to identify such typical changes following a motor to mental gradient of skills, which affects neuronal processes.

We have seen, across each domain of expertise, that the degree of functional brain plasticity reflecting an increased level of expertise was task-dependent and followed extensive practice. Enhanced behavioral and cognitive performances involve dynamic shifts in the strength of pre-existing neuronal connections, including changes in task-related cortico-cortical and cortico-subcortical coherence. In the motor domain, experts usually yield a reduced pattern of brain activity in the cortico-cerebellum pathway, despite a more focused activation in the striatum (caudate nucleus/putamen), suggesting that this structure may be critical for long-term retention of well-learned motor sequences. Likewise, in the cognitive domain, Dahlin et al. (2009) demonstrated positive behavioral effects of training on working memory which are associated by a decrease in cortical areas typically related to working memory along with fewer peaks of activity in regions participating to attentional control processes (e.g., fronto-parietal regions) and increases in the striatum. Together, these data suggest that following a motor to mental gradient, there is lower involvement of control-related cortical areas (e.g., prefrontal cortex) and an increase in the recruitment of the striatum along with practice. Accordingly, we also underlined in this review the role of basal ganglia across the motor to mental gradient. Yet, basal ganglia are not restricted to motoric aspects of behavior, but rather involved most areas of cognitive and emotional functioning (Seger, 2006), which is consistent with their anatomical connections with all areas of the cortex (Alexander et al., 1986). Briefly, basal ganglia interact with the cortex through independent processing loops in which the cortex projects to the striatum, the striatum to the pallidum, the pallidum to the thalamus, and from there back to the cortex. These processing loops have functions that complement those of the cortical areas they interact with. Neuroimaging studies in humans have undoubtedly demonstrated that basal ganglia play a critical role in the planning, learning, and the execution of a new motor skill, as well as the long-lasting representation of the skilled behavior (Doyon et al., 2009). In the same vein, and in addition to common activations in brain areas associated with generation and maintenance of mental images in the working memory, basal ganglia showed increased activity after intensive motor imagery practice, and therefore demonstrate its involvement in the expertise level of performance and reinforcement learning (Baeck et al., 2012). Finally, the contribution of basal ganglia in meditation has also been reported, albeit in a different way, in experienced meditators. Indeed, meditative state starts by activation of frontal regions that would successively activate, in cascade, different cortico-thalamic-limbic-basal ganglia loops that would maintain the meditative state. Ritskes et al. (2003) further demonstrated that when experienced meditators switched from normal consciousness to meditative state, increased activation in the prefrontal cortex and basal ganglia occured along with decreased activation in the superior occipital gyrus and anterior cingulate. It has been hypothesized that increased activations may be associated with the gating of cortical–subcortical interactions that leads to an overall decrease in readiness for action (Kjaer et al., 2002).

As seen in this review, complex motor skills that involve procedural learning, as well as purely mental skills, result in measurable changes in brain structure. Recently, studies using MR-based morphometry provided new insight to our understanding of brain plasticity related to the expertise level. Accordingly, clear modifications of the gray and white matter volumes are related to the skill level, which in turn depends on training characteristics. Moreover, those structural changes have been shown to correlate with behavioral improvements (for motor practice) and attentional and emotional regulation (for meditation). This latter finding might serve as a neural body of evidence for the common idea that longer practice makes perfection. However, it remains unknown when morphometric changes can first be detected and how long changes last.

One of the main characteristic that entails neural functional plasticity across a motor to mental gradient of expertise is that experts achieve higher levels of performance with less cognitive effort. Especially, using a quantitative ALE meta-analytic method, Patel et al. (2013) demonstrated common reduced activation for both motor and cognitive training in regions closely overlapping with the fronto-parietal control and dorsal attention networks. Indeed, it remains difficult and time-consuming to control multiple tasks concurrently (Miller, 1956; Just et al., 2001; Rubinstein et al., 2001), so that any strategy that minimizes the demands placed on intentional control is of great benefit. For instance, the functional activation observed in premotor areas or M1 during motor performance is reduced or more focused in professional musicians compared to amateurs or non-musicians (Hund-Georgiadis and von Cramon, 1999; Meister et al., 2005). More generally, the reduced activation in highly skilled performers is often taken as evidence for “increased efficiency of the motor system” and the need for a smaller number of active neurons to perform a given set of movements (Krings et al., 2000; Haslinger et al., 2004), albeit others researchers argued that lower levels of activation result from reduced attention or task difficulty (Floyer-Lea and Matthews, 2004; Landau and D’Esposito, 2006). As suggested by Picard et al. (2013), low activation is not always a sign of low neuronal activity, but it may rather be a reflection of plastic mechanisms involved as expertise emerges. Connectivity studies provide a definitive answer to this point by examining whether the reduction in activity of certain areas is followed by increased activity of other brain regions when experts perform skilled and non-skilled tasks.

## FUTURE PERSPECTIVES

In this review, we attempted to provide an overview, although not exhaustive, of structural and functional brain plasticity that occurs while practicing at an expert level of performance. It is worth acknowledging that the emphasis given on each specific, albeit substantial, domain of research following a motor to mental gradient, imposes limits given that each constitutes a currently very active area of research in cognitive neuroscience. Nevertheless, and despite these limitations, the main purpose of this review was to select and consider the most relevant insights in the three domains of expertise and to identify the common patterns of brain changes.

As a postulate, changes in the functional or structural networks may be expected to occur after long-term intensive skill training. Many studies exploited those changes through cross-sectional designs to reveal group differences underpinning expert skills of performance. However, the main issue using this approach is the difference in individual anatomical networks before training that could allow for predisposition in practicing a specific skill. A solution to this problem comes from longitudinal studies, which are time-consuming and often nearly impossible if training occurs over long time periods. Thus, in the future, the emphasis should be the use of multimodal imaging approaches (e.g., DTI and high-field MRI) to provide conjoint analyses of changes in activity within specific regions and patterns of connectivity between different regions. Further work is still needed to better discriminate the specific composition of training tasks that influence expertise and how/when this is reflected in functional and structural plasticity.

Although further research is needed to understand and identify the functional and structural changes protracted by expertise, the current challenge to modulate neural plasticity for optimal and long-term behavioral gains is now possible. The use of real-time fMRI specifically opens a space to examine functional changes associated with the acquisition of a motor or cognitive skill, since participants have been educated to gain some familiarity with the neuroanatomy before neurofeedback sessions (Sulzer et al., 2013). Given that brain-activation patterns and behavior are assumed to be closely linked, it seems likely that learned control over brain activation would lead to changes in cognition and behavior (Fetz, 2007). A growing research is actually attempting to develop new types of task paradigms that might benefit most from neurofeedback fMRI. This neuroimaging technology has already demonstrated performance-enhancement applications, such as boosting memory by increasing activation in memory-related brain areas (Zhang et al., 2013), or improving motor imagery accuracy (de Charms et al., 2004). Likewise, non-invasive brain stimulations such as transcranial magnetic or direct current stimulation (TMS/tDCS) induce lasting effects that can be used to explore the mechanisms of cortical plasticity in the intact human cortex and determine therapeutic potentials for behavioral and cognitive improvement. For example, Marshall et al. (2004) showed that the application of tDCS over frontal areas during slow-wave sleep contributed to improve declarative memory consolidation. Thus, both approaches can be applied to explore and enhance brain plasticity in the context of expertise, and future research should certainly investigate the synergist effects with sleep homeostatic functions. Enthusiastic findings have already demonstrated that rTMS applied concomitantly to daily motor training during 4 weeks might contribute to improve motor performance, along with corresponding neural plasticity in motor sequence learning networks (Narayana et al., 2014).

## Conflict of Interest Statement

The authors declare that the research was conducted in the absence of any commercial or financial relationships that could be construed as a potential conflict of interest.
