# Corrigendum: Experts bodies, experts minds: how physical and mental training shape the brain

**DOI:** 10.3389/fnhum.2015.00047

**Published:** 2015-02-09

**Authors:** Ursula Debarnot, Marco Sperduti, Franck Di Rienzo, Aymeric Guillot

**Affiliations:** ^1^Département des Neurosciences Fondamentales, Centre Médical Universitaire, Université de GenèveGenève, Switzerland; ^2^Centre de Psychiatrie et Neurosciences (InsermUMRS894), Université Paris DescartesParis, France; ^3^Laboratoire Mémoire et Cognition, Institut de PsychologieBoulogne Billancourt, France; ^4^Centre de Recherche et d'Innovation sur le Sport, University Claude Bernard Lyon 1Villeurbanne, France

**Keywords:** expertise, meditation, motor imagery, motor skills, motor consolidation, neural networks

An important reference (Yarrow et al., 2009) has been mistakenly omitted from the published article.

The review uses the material of the article by Yarrow et al. in three parts, specifically in the section: The neurocognitive basis of motor skill learning. The sentences were, unfortunately, used and cited verbatim without citing this important reference. The passages concerned are reproduced below.

“In cognitive psychology, theoretical descriptions of changes in skilled performance were shown to move from cognitive to automatic processing (Fitts, 1964). The key concept is the increasing automaticity: controlled processes are attention demanding, conscious and inefficient, whereas automatic processes are rapid, smooth, effortless, require little attentional capacity, and are difficult to be consciously disrupted (Shiffrin and Schneider, 1977).”

“Practically, it is not automaticity per se that is indicative of high proficiency, but rather the level of skill at which automaticity is attained. Although the border between the automaticity and the expertise concepts beg for clarification, one may consider that most people fail to develop beyond a hobbyist level of performance as they settle into automaticity at a given level of skill that they find enjoyable, rather than continuing to improve skills (Ericsson, 2007). Hence, automaticity is more a false ceiling than a measure of excellence.”

“One solution to this issue is through assessment of changes in speed-accuracy trade-off functions, i.e., to defy the speed–accuracy tradeoff for a given task (Krakauer, 2009; Krakauer and Mazzoni, 2011). In other words, a skilled tennis player can serve both faster and more accurately than a novice. Thus, sporting skill at the level of motor execution can be considered as acquiring a new speed–accuracy trade-off relationships for each sub-task of the motor sequence.”

These sentences are lifted verbatim from the study by Yarrow and collaborators in their important review paper, which should have therefore been cited explicitly. This is an individual error that we collectively take responsibility for. The authors apologize for their oversight and the unintentional inconvenience caused.

## Conflict of interest statement

The authors declare that the research was conducted in the absence of any commercial or financial relationships that could be construed as a potential conflict of interest.
